# The role of the cytoskeleton and molecular motors in endosomal dynamics

**DOI:** 10.1016/j.semcdb.2014.04.011

**Published:** 2014-07

**Authors:** Elizabeth Granger, Gavin McNee, Victoria Allan, Philip Woodman

**Affiliations:** Faculty of Life Sciences, University of Manchester, Manchester M13 9PT, UK

**Keywords:** Endosome, Lysosome, Recycling, Dynein, Kinesin, Myosin

## Abstract

The endocytic pathway is essential for processes that define how cells interact with their environment, including receptor signalling, cell adhesion and migration, pathogen entry, membrane protein turnover and nutrient uptake. The spatial organisation of endocytic trafficking requires motor proteins that tether membranes or transport them along the actin and microtubule cytoskeletons. Microtubules, actin filaments and motor proteins also provide force to deform and assist in the scission of membranes, thereby facilitating endosomal sorting and the generation of transport intermediates.

## Highlights

• Transport between endocytic organelles involves microtubule and actin based molecular motors.
• Both cytoskeletal systems contribute to membrane deformation and scission.
• Transport of endosomes to and from the cell cortex requires transfer between actin and microtubules.

## 1 The endosomal system: an overview

**Fig. 1:**
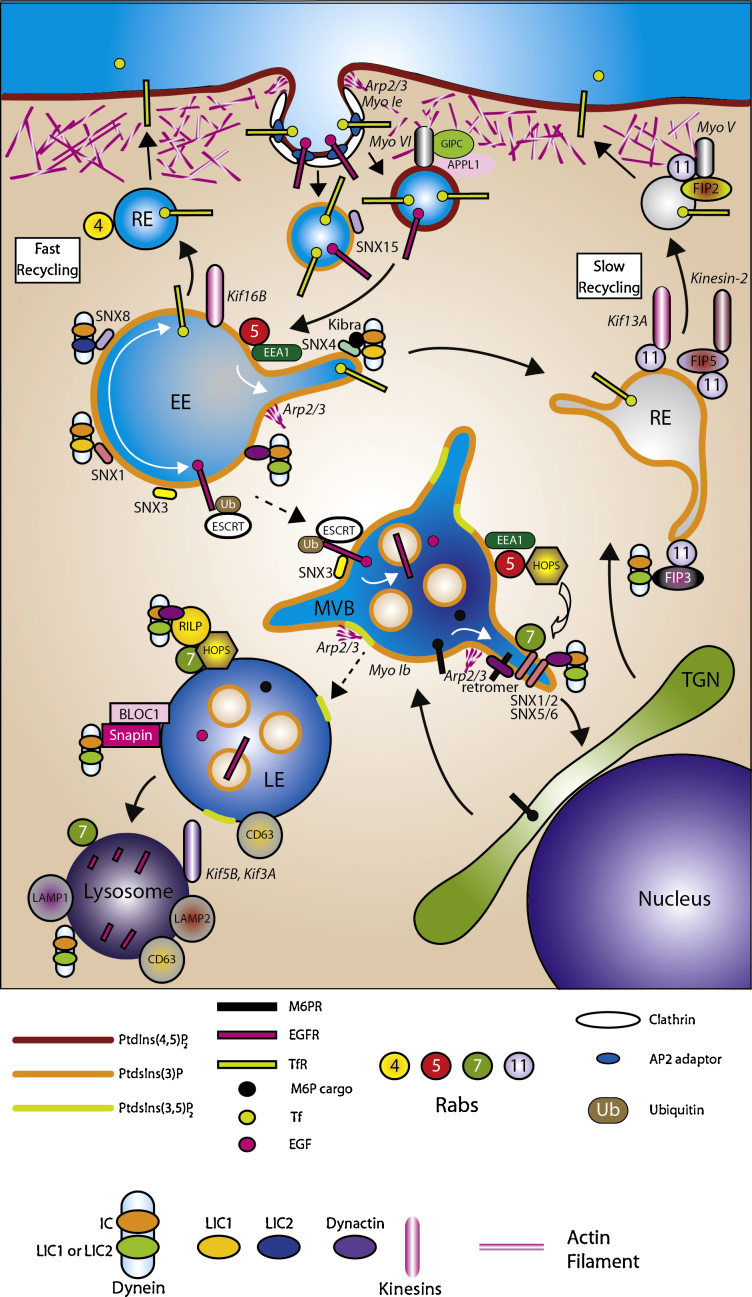
Overview of the endocytic pathway and the cytoskeleton. Transport steps are shown using solid arrows, maturation steps are shown using broken arrows.

The early endosome, characterised by the presence of rab5 and EEA1, acts as the major sorting station on the endocytic pathway (Fig. 1). Several populations of endocytic vesicle deliver content to the early endosome from the plasma membrane. At least two of these are generated by a clathrin-dependent pathway; APPL1-positive [1] and SNX15-positive [2] vesicles. The early endosome also receives vesicles derived from the TGN. From the endosome, cargo is sorted: for degradation, via a pathway comprised of multivesicular bodies (MVBs), during which endosomes mature and then fuse with the lysosome; or for recycling back to the plasma membrane or TGN [3]. Recycling to the plasma membrane occurs via slow or fast pathways. “Fast” recycling takes material directly from an early endosome back to the plasma membrane, and is regulated by Rab4 and Rab35 [4,5]. In contrast, “slow” recycling transits through Rab11-positive recycling endosomes [6–8]. The major pathways and important molecular components are shown in Fig. 1. How each motor associates with various endocytic compartments and contributes to endocytic function (detailed below) is summarised in Table 1.

## 2 Motor proteins

Microtubules support intracellular transport in a manner that is dependent on their intrinsic polarity. They possess a dynamic plus end that, in most cells, grows towards the periphery and a minus end that is anchored in the microtubule organising centre, normally located near the nucleus in non-dividing cells. Microtubules support both short- and long-range movements, using two types of motors: kinesins, with a few exceptions, move towards plus ends [9], whilst cytoplasmic dynein (henceforth called dynein) moves towards minus ends [10].

**Fig. 2:**
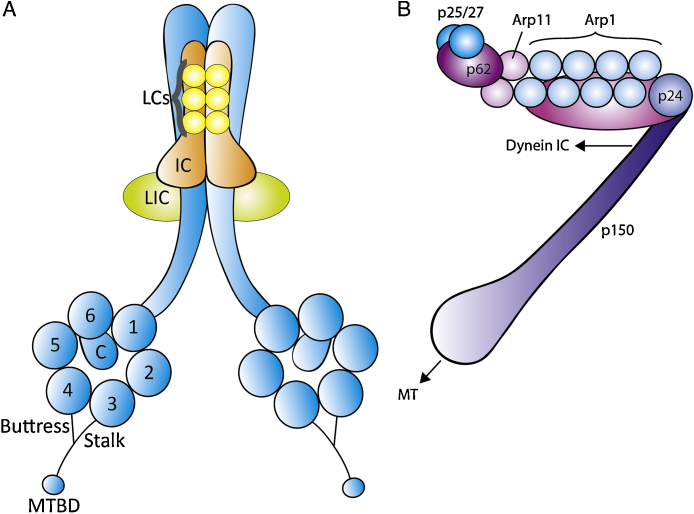
Molecular composition of dynein (A) and dynactin (B). The AAA ATPase subunits (1–6) and C-terminal domain (C) of dynein heavy chain (blue) are labelled.

Dynein is a large (1–2 MDa) motor complex (reviewed in [10]) that assembles around 2 copies of dynein heavy chain (DHC) (Fig. 2A). DHC contains a motor domain, and a tail domain that binds two copies each of intermediate chain (IC) and light-intermediate chain (LIC). IC in turn binds 3 types of light chain (LC; TCTEX, LC8 and Roadblock/LC7). Two alternate genes encode each of these accessory subunits, and IC and LIC produce a range of differentially spliced isoforms, so generating dynein complexes of considerable heterogeneity. Dynein interacts with several regulators, including three involved in most, if not all, of dynein's cellular functions: lissencephaly 1 (LIS1), nuclear distribution E (NUDE, of which there are two related genes, Nde1 and Ndel1, in mammals) and the dynactin complex (Fig. 2B) [11].

Kinesins consist of a large superfamily [12,13]. The plus-end-directed kinesins that drive endosome motility are mostly members of the kinesin-1, kinesin-2 and kinesin-3 families (Table 1; [12,14]). Kinesin-1 contains a homodimer of motor subunits, with KIF5B being ubiquitously expressed while KIF5A and C are primarily neuronal. These associate with two light chains, which are encoded by two genes, one of which is extensively alternatively spliced. Kinesin-2 can be heterotrimeric with two motor subunits (KIF3A paired with KIF3B or KIF3C) and an accessory subunit, KAP3. Other family members, such as KIF17, form homodimers [12,14]. The kinesin-3 family has many members, which may act as monomers or dimers. Other kinesins with roles in endosome movement include the kinesin-13 KIF2β, and the minus-end-directed KIFC1 and 2 (see Table 1).

Actin filaments also display polarity, with barbed and pointed ends analogous to the plus and minus ends, respectively, of microtubules, and generally support short-range movements in animal cells. The actin-based motors, myosins, share an evolutionarily conserved core mechanism with kinesins [15], and also belong to a superfamily [14]. Myosins walk to the barbed end of the filament, with the exception of myosin VI [9]. Those families most relevant to endocytic trafficking are myosins I, V and VI. Polarised actin polymerisation on the surface of organelles to generate actin ‘comets’ can also support organelle movement [16].

## 3 Uptake from the plasma membrane: the role of actin

Uptake occurs through multiple routes, including clathrin-dependent endocytosis, caveolae, phagocytosis, macropinocytosis and several other clathrin-independent pathways (reviewed in [17]). Actin has a particularly well-characterised role during phagocytosis (not a subject of this review, but see [18]) and clathrin-dependent endocytosis [19]. In yeast, actin is absolutely required for clathrin-dependent endocytosis unless the turgor pressure across the plasma membrane is reduced experimentally [20]. In mammalian cells, actin is not always essential. Its importance is more prominent when a larger force is required for budding, such as endocytosis at plasma membrane locations that display a high degree of membrane rigidity [21], or during the ingestion of larger cargoes [22]. Hence, actin has a clear role in generating force to assist uptake.

During endocytosis, actin polymerisation with the barbed ends pointing towards the membrane helps to stabilise and elongate the newly formed neck of a nascent vesicle by exerting force against the membrane [19,23]. Type I myosins combine with verprolin and WASP (Wiskott-Aldrich syndrome protein) family proteins to activate the Arp2/3 complex, which in turn drives actin polymerisation [24,25]. These myosins are recruited to sites of endocytosis by elements of the endocytic machinery. For example, myosin 1e binds the scission protein dynamin and the phosphatidylinositol (PtdIns)-5 phosphatase synaptojanin-1 [26]. Since they can also bind to membrane lipids whilst associated with actin filaments [27], type I myosins may also help to generate membrane tension and local membrane deformation [26,28]. Furthermore, myosin Ie [25] and actin polymerisation [23,29] may support the movement of endocytic vesicles away from the plasma membrane.

In mammalian cells the minus end-directed myosin VI is recruited to sites of clathrin-dependent endocytosis [30]. Contrasting with the function of type I myosins in modulating actin organisation and polymerisation, force generated by myosin VI can directly contribute to vesicle formation, as demonstrated for a tissue-specific splice variant (myosin VI-LI) at the apical surface of polarised cells [31]. Here it interacts with the endocytic adaptor, disabled homologue 2 (DAB2) [32,33].

## 4 Transport away from the cell cortex

Newly formed endocytic vesicles are transported away from the cortical region as they switch from actin- to microtubule-based movement, with dynein being the major minus end-directed microtubule motor (see below). A specific isoform of myosin VI (NI; no insert splice variant) assists the transfer of nascent endocytic vesicles away from the actin-rich cell periphery in preparation for their delivery to the microtubule transport system [34]. One crucial myosin VI adaptor acting here is GIPC [35], which links myosin VI to APPL1, a marker for a peripheral population of Rab5-positive early endosomes that act upstream of the EEA1-positive sorting endosome [1,36–38]. A further myosin VI adaptor, Tom1 (or Tom1L2) also associates with APPL1 endosomes [39], though this interaction seems more important for generating autophagosomes.

One protein that may regulate the switch from actin- to microtubule-based movement of endocytic compartments is Huntingtin (Htt), in which expansion of poly-glutamine repeats leads to Huntington's disease [40]. Htt localises to peripheral vesicles and partially colocalises with clathrin [41]. Moreover, the Htt-binding proteins Htt-interacting protein 1 (HIP1) and HIP1R link clathrin to the actin cytoskeleton and are recruited to nascent coated vesicles concomitant with myosin VI [30]. Htt interacts directly with dynein IC [42], and also indirectly via Htt-associated protein 1 (HAP1) with both kinesin-1 and dynactin [43,44], and disruption of Htt causes peripheral accumulation of all endocytic compartments, consistent with impaired dynein-dependent trafficking [45]. For one endocytic organelle studied in detail – the late endosome/lysosome – it was seen that dynein depletion led to the lysosomes being enmeshed in cortical actin, but that this did not occur when Htt was depleted as well [45]. Although not directly tested for early endocytic vesicles, this suggests that Htt could help link cargo to actin and coordinate switching between actin and microtubule cytoskeletons, in preparation for dynein-dependent movement. Providing further support for such a model, another Htt-binding protein, HAP40, forms a ternary complex with Htt and Rab5, and promotes the association of early endosomes with actin rather than microtubules [46]. Moreover, cells overexpressing HAP40 or containing a mutant Htt exhibit increased binding of endosomes to actin, impaired endosome motility, and reduced transferrin (Tf) uptake [46]. It is important to note that switching of endosomes between actin filaments and microtubules is not restricted to the cell cortex [46,47], and is regulated by additional factors such as Rho GTPases and formins [48].

## 5 Endosome motility

It is well established that all types of endosome move actively along microtubules. This is particularly clear in neurons, but extensive motility is also seen in cultured cells such as HeLas. High frame rate imaging and automated particle tracking has recently provided insight into these dynamics.

Rab5-positive early endosomes move with an inward bias [49,50], but can also move outward and occasionally bidirectionally [49]. Their motile behaviour is governed to some extent by cellular location, since endosomes localised to peripheral actin-rich zones tend to move only short distances, as might be expected [49]. Early endosome motility is clearly highly complex, since individual endosomes can stop and start, and move for long or short distances, wherever they are in the cell. Systematic analysis of endosome motility employing fast imaging (up to ∼30 fps) [51–53] provides quantitative description of such behaviour, showing that about half of Rab5-positive endosomes undergo directed motion during a 30–50 second recording [52,53]. Whilst most movements are short-range (i.e. <1 μm), some occur over several microns. Approximately 80% of such long movements are dynein-driven translocations towards the cell centre, exhibiting average speeds up 1–2 μm s^−1^, and peak observed speeds often of 4–6 (and occasionally as fast as 8) μm s^−1^ [52,53].

Long-range movements occur in short bursts of directed motility (*runs*), interspersed with somewhat longer periods (*rests*/*pauses*) of non-directed (i.e. diffusive) motion, and with occasional reversals [52,53]. It is likely that these abrupt changes in motile behaviour generate high membrane tension and are linked to events such as cargo sorting and membrane fission [50,52–54]. Run terminations occur when microtubules cross over, or when endosomes encounter each other [52,53]. Surprisingly, disrupting the actin network does not markedly influence endosome *run*/*rest* behaviour, but runs often terminate when endosomes contact ER tubules [53]. These endosome-ER encounters, also observed at the EM level [55], can be relatively long-lived, and tension in the ER tubule is often evident [53]. In all, the complex motility of endosomes, coupled to their rapid fission and fusion [54], allows the early endosomal system to generate a highly dynamic network of membranes in keeping with its key function of exchanging and redistributing cargo throughout the cell [56]. Rab7-positive late endosome motility has also been observed at high frame rate, and these display significantly more bidirectional movement than early endosomes [53,57].

### 5.1 The molecular basis for dynein-driven endosome/lysosome motility

The inward movement and localisation of all endocytic compartments is determined largely by dynein, since inhibition of dynein causes scattering of early endosomes, late endosomes and lysosomes and disrupts endosome motility [50,52,53,58–61]. Furthermore, dynein is present on endosomes [62–65], and disruption of dynein activity inhibits early [64] and late [65] endosome motility *in vitro*. However, minus end-directed kinesin(s) may be important in some circumstances [66,67].

It has been proposed that dynein-dependent movement is initiated by a cascade of protein interactions located at the plus ends of microtubules that ‘capture’ the endocytic vesicle and then activate motility. Several proteins (termed +TIP proteins) including EB1, EB3 and CLIP-170 accumulate at the plus ends of growing microtubules where they regulate microtubule dynamics and interactions with the cell cortex [68]. Recruitment of dynactin to microtubule plus ends via binding of the CAP-Gly region of p150^*Glued*^ to CLIP-170 [69] could in turn recruit and activate dynein in the vicinity of cargo [61,70]. Notably, Lis1 is also concentrated at microtubule plus ends and, at least in filamentous fungi, has been shown to initiate dynein-dependent cargo movement [71,72], so might assist in this activation pathway [68]. The p150^*Glued*^ dynactin subunit can also bind to microtubules directly via its CAP-Gly and basic regions [73,74], independently of other +TIP proteins, and this interaction has been proposed to enhance dynein's ability to take many steps along the microtubule before detaching [74,75].

However, whilst the dynactin-mediated microtubule ‘capture’ pathway may be important for activating the transport of endosomes and other organelles from microtubule plus ends in the distal regions of neurons [76–78], as well as for the rapid activation of melanosome movement [79], it is not obligate for dynein-dependent organelle movement in most contexts. Preventing dynactin from binding to microtubules or to +TIPs does not cause endosomes to accumulate in the cell periphery [80] or impair organelle movement [80–82] in non-neuronal cells, and does not prevent endosome or lysosome movement in the mid-axon region of neurons [76–78]. Thus, +TIP or microtubule ‘capture’ may be important under particular circumstances, but independent mechanism(s) must operate to load cargo and dynein elsewhere on the microtubule more generally. A somewhat similar situation may exist in filamentous fungi such as *Ustilago maydis* and *Aspergillus nidulans*, where dynein supports the retrograde movement of early endosomes away from the hyphal tip, whilst kinesin-3 moves them to the tip [83,84]. A locally high concentration of dynein at the microtubule plus end, maintained by a combination of +TIP proteins and kinesin-1 dependent outward movement of dynein [71,85], helps prevent endosomes falling off the microtubule plus end [86] and is thus important for maintaining this high-flux pathway. However, it is not obligate for activating dynein on endosomes [87], an event that can also occur mid-microtubule [88].

Endocytic compartments use a range of mechanisms to engage this multisubunit motor (Table 1). Surprisingly, molecular details of how dynein binds early endosomes are limited, though Rab5 and dynein do co-immunoprecipitate [89], and Rab4 binds directly to LIC1 [90]. Additionally, a neuronal-specific isoform of IC, IC1B, is found on dynein transporting TrkB signalling endosomes [91]. Meanwhile, studies in *A. nidulans* [92] and more recently in mammalian cells [93] have shown that the dynactin p25 subunit is needed for dynein-early endosome association. One route through which this may act is via Hook, a conserved microtubule-binding protein that in *A. nidulans* and *U. maydis* links the dynein-dynactin complex to early endosomes [94,95].

Dynein/dynactin are linked to late endosomes/lysosomes via Rab7-interacting lysosomal protein (RILP) and Oxysterol-binding protein-related protein-1L (ORP1L), which bind Rab7 to generate a membrane-bound tripartite complex [96,97]. Here, RILP is thought to interact with dynactin p150^*Glued*^ [96] and LIC1 [63] to load dynein onto the organelle, whilst ORP1L is necessary for ensuring that the motor is active [96]. Importantly, the interaction between ORP1L and RILP is regulated by cholesterol; when cholesterol levels are low, ORP1L adopts a conformation that favours an interaction with an ER membrane protein, vesicle-associated membrane protein (VAMP)-associated ER-protein (VAP). This generates ER contacts that retain late endosomes in the cell periphery [98]. Interestingly, RILP binds to the HOPS complex [99], which is involved in late endosome-lysosome fusion [98], whilst associated with dynactin. Hence, RILP and ORP1L may link late endosome motility with delivery of cargo to lysosomes.

Further routes for recruiting dynein to lysosomes have been identified recently. In Zebrafish neurons, the single LIC interacts with c-Jun N-terminal kinase interacting protein 3 (JIP3), to mediate the retrograde transport of lysosomes [100]. Snapin, a component of the Biogenesis of Lysosomal Organelle-like Complex-1 (BLOC-1) that is required for late endocytic trafficking and the generation of melanaosomes and other lysosome-related organelles [101], interacts with IC and is needed for dynein-driven late endosome retrograde transport, at least in neurons [102]. Interestingly, the IC-Snapin interaction, and recruitment of dynein to late endosomes, is reduced in the brains of mice expressing disease-associated mutant human Alzheimer's precursor protein (APP). This results in reduced lysosomal degradation, and hence higher levels, of β site APP-cleaving enzyme 1 (BACE), the major β secretase that generates β-amyloid [103]. Finally, the Rab4 effector, Rabip4′, and the AP3 clathrin adaptor complex (known to be important for lysosomal trafficking) associate with dynein and are important for maintaining lysosomes centrally in the cell [104]. In summary, several interactions between dynein and membrane-associated proteins have been identified which may help recruit dynein within the endocytic system, but further work is needed to understand how these work together to coordinate the inward movement and transport of cargo.

### 5.2 The molecular basis for outward microtubule-based endosome/lysosome motility

Both kinesin-1 and -2 move early endosomes in vitro [64,105], while in vivo studies have highlighted a role of kinesin-3 family members in transporting Rab5-positive endosomes in *Drosophila* [106], *U. maydis* [83,84] and *A. nidulans* [83,84]. Meanwhile, in mammalian cells, Rab5-positive endosomes also move to the cell periphery using a kinesin family-3 motor, KIF16B [107]. KIF16B interacts with the early endosomal lipid PtdIns-3P via a Phox homology (PX) domain in its tail region, and is thought to control the balance between receptor recycling and degradation by maintaining internalised cargo close to the plasma membrane and away from the degradative pathway [107]. KIF16B also acts during the transcytosis of the transferrin receptor (TfR) in epithelial cells [108].

Plus end-directed transport of late endosomes and lysosomes is driven by several kinesins. Hence, whilst disruption of the kinesin-2 KIF3A motor subunit induces the clustering of both late endosomes and lysosomes [109], and KIF3A moves late endosomes *in vit*ro [65], the kinesin-1 motor KIF5B is required for the redistribution of lysosomes to the cell periphery induced by cytoplasmic acidification [110–112]. Recruitment of kinesin-1 via its light chains to lysosomes is mediated by SKIP (SifA and kinesin-interacting protein) [113,114], which in turn binds the lysosome-associated Arf-like GTPase, Arl8 [113]. Meanwhile, other studies have implicated a splice variant of the kinesin-3 family member KIF1Bβ [115] and a kinesin-13 family motor, KIF2β [116], in promoting plus end-directed lysosomal transport. The reason for such a spectrum of kinesins contributing to lysosome motility (summarised within Table 1) is not clear. It may point to subpopulations of lysosomes whose positioning is regulated differentially, or to cell type-specific pathways.

### 5.3 Bidirectional movement of endocytic compartments

Endosomes moving along microtubules do on occasion switch directions, suggesting that at least a portion carry both plus- and minus-end-directed microtubule motors. Switching is fairly rare for early endosomes [52,53], but common for LE/lysosomes [57]. Furthermore, bidirectional movement plays an important part in the dynamics of recycling endosomes (Section 6.1) [58].

Although the mechanism by which bidirectional transport can occur [117] is still not fully understood, opposing motors present on the organelle must either be switched on and off in a co-ordinated manner, or must maintain (at least for a period) a competitive, “tug-of-war” balance of forces that can be tilted to favour either direction [118,119]. Evidence that endosomes can switch direction based on motor competition has been observed in *Dictyostelium* and in HeLa cells [120], where early endosomes moving in one direction can pause and undergo intermittent elongation, as if being subjected to opposing forces, before reversing direction. In *Dictyostelium* at least, several, weaker, dynein motor units balance each kinesin [120]. A “tug-of-war” on endosomes is also observed in *U. maydis*, though here dynein appears to be the stronger motor [88]. Here, the balance of forces may also be influenced by the action of Hook; this dynein adaptor also binds to kinesin-3, and appears to reduce the amount of the plus-end motor bound to outward-moving endosomes just as they meet inward-moving dynein on the microtubule [95]. Of course, endosomes/lysosomes might also under certain circumstances employ coordinated switching of motor activities to alter direction, as has been observed for other organelles [121]. Indeed, observations in COS7 cells show that lysosomes can switch direction either rapidly or more gradually, and acute disruption of dynein function causes decline in outward lysosome movement, after a transient increase [57].

Such behaviour suggests that some form of coordinate regulation is in play. Htt is implicated in controlling the direction of movement of neuronal endosomes, since Htt phosphorylation by Akt stimulates plus-end-directed movement, while retrograde movement predominates in the presence of dephosphorylated Htt [122]. The direction of recycling endosome movement, on the other hand, can be controlled by JIP3/4. JIPs have previously been implicated in microtubule-based motility, since they bind to kinesin-1 [123–125], and to dynactin [126,127] to regulate both minus- and plus-end-directed microtubule transport. On the recycling endosome, switching of JIP3/4 between kinesin-1 and dynactin is regulated by the small GTPase ARF6 [127]. Hence, Htt and JIPs may act more widely as focal points for regulating microtubule-based movement of endocytic compartments.

## 6 Endosomal sorting and recycling

Dynein is important for sorting recycling cargo such as Tf away from degradative cargo such as EGF [50]. Such sorting may involve the segregation of endosomal membrane domains by means of a “tug-of-war” between dynein and a counteracting kinesin, as described above [120]. However, dynein also supports the sorting of recycling cargo into tubules that emanate from endosomes at various points during their maturation to late endosomes. These tubules are generated by sorting nexins (SNXs), a family of membrane-deforming proteins that transport many proteins to the plasma membrane or TGN, often in cooperation with the Vps26/29/35 cargo-binding retromer complex [3]. Each SNX acts in a subset of sorting events. For example, efficient recycling of mannose-6 phosphate receptors from later endosomes to the TGN requires retromer and SNX1/2, together with SNX5/6 [3]. Here, SNX5/6 recruits dynactin to mediate dynein-dependent tubule elongation and/or scission [128]. Meanwhile, SNX4 mediates the sorting of TfR from the early endosome to the Rab11-positive recycling endosome, and this process is aided by the adaptor protein KIBRA binding to both SNX4 and dynein LC8 [129]. Recent work adds a layer of complexity, by showing that the motility of SNX1- and SNX4-positive membrane regions in early endosomes depends on dynein containing LIC2, whilst those positive for another sorting nexin, SNX8, use dynein with LIC1. Moreover, depleting dynein impairs the normal segregation of SNX1/4 and SNX8 into distinct regions of the endosome [58]. Interestingly, depleting the KIF5B subunit of kinesin-1 inhibited long-range movement of SNX1 and SNX8, whereas depleting the kinesin-2 KAP3 subunit reduced SNX4 motility [58]. Hence, different subsets of motors could direct the formation of distinct tubules with differing SNX composition.

Dynein is required not only for the generation of recycling intermediates but also for maintaining the position of the recycling endosome itself. This is achieved at least in part by the binding of LICs to the Rab11 effector FIP3 [130,131]. Perhaps counter-intuitively, the kinesin-3 member KIF13A also directs the formation of TfR-positive recycling tubules from the sorting endosome, supports efficient TfR recycling, and interacts directly with Rab11 family members to generate a motile tubular recycling endosome [132]. Moreover, kinesin-2 also supports TfR passage through the recycling endosome via an interaction with the Rab11 effector FIP5/RIP11 [133], whilst kinesin-1 transports TfR from the TGN to the plasma membrane via the recycling endosome, involving interactions with Gadkin and AP1 adaptor proteins [134]. Finally, kinesin-1 contributes to the localisation of the recycling endosome in the *C. elegans* gut epithelium [135]. The finding that both plus- and minus-end directed microtubule motors help cargo transit to the recycling endosome is not surprising, however, given the pleiomorphic nature of this compartment, which in some cells is focussed around the MTOC [8] but is often more peripheral. This differential localisation correlates to some extent with levels of stable microtubules in different cell lines. In those where the recycling endosome is centrally located, subsequent transfer of TfR to the periphery is kinesin-dependent (though the family member undefined) [136]. As mentioned above, switching of Rab11 between kinesin-1 and dynactin is controlled by ARF6 in conjunction with JIP3/4 [127].

The generation of tubular sorting intermediates at the endosome is also assisted by actin [137]. Actin assembly into branched polymers is regulated by the ARP2/3 complex [138], which in turn is activated at specific membranes by WASP family members. At the endosome, WASH (Wiskott-Aldrich syndrome protein and SCAR homologue) binds to retromer and recruits Arp2/3 to generate cargo-specific, actin-rich patches that may assist in the generation and/or scission of recycling tubules formed by SNXs (see [139] for an excellent review on the WASH complex). Interestingly, the WASH complex also binds to tubulin [140], and thus may promote static endosome-microtubule interactions that ultimately lead to microtubule motor engagement. Arp2/3-dependent actin assembly on endosomes can also be directed independently of WASH, via annexin A2 and Spire1 [47]. Here, this actin polymerisation plays an important role in allowing the vacuolar region of the early endosome (the endosomal carrier vesicle or MVB) to fully develop and deliver cargo efficiently to the lysosome, with branched actin polymers perhaps separating recycling tubules away from the developing MVB [47].

In addition to the role of actin polymerisation in promoting membrane deformation, separation of membrane domains and carrier biogenesis, actin motors are also prominent at the endosome. Myosin VI acts with LMTK2 (lemur tyrosine kinase 2) to help TfR exit the sorting endosome and reach the Rab11-positive recycling endosome [141]. Here, myosin VI may perform functions akin to those described for it at the plasma membrane (see above). Myosin-Ib also has an important though not fully defined role in the transport of cargo from endosomes to lysosomes, perhaps helping to tether membranes together, or coordinating long-range lysosome motility [142–144].

In summary, microtubules and their associated motor proteins function in concert with actin assembly and myosins to promote membrane tubulation and endosomal maturation. The mechanical forces exerted by these components may ultimately combine to induce membrane tension at critical points in the process in order to generate, elongate and break membrane tubules. Clearly, however, much work is required to clarify how these molecular events are ordered.

## 7 Transfer to the cell surface

Once cargo has been transported through the recycling endosome and has reached the cell periphery, transport to the plasma membrane is facilitated by the action of the actin cytoskeleton and its associated motors, principally myosin Vb. Type V myosins are widely implicated in carrier transit to the cell surface. Consistent with this, myosin V isoforms bind multiple Rabs involved in exocytotic processes, including Rab3, Rab27A, Rab8 and Rab10 (see [145] and references therein). Whether myosin Vb promotes recycling by supporting long-range movement or by acting at short range within the cortex has been a subject of much study. Myosin V directly moves cargo to the peripheries of specialised structures that possess clearly oriented actin organisation, such as dendritic spines [146–148]. However, quantum dot-labelled myosin V moves only in very short directed runs within the cortical region of COS-7 cells where the actin cytoskeleton is poorly ordered [149].

It remains unclear how such short-range motility contributes to the transport of cargo over large distances [150,151]. Myosin Vb binds Rab11 [152] and Rab11-family-interacting protein-2 (Rab11-FIP2) [153]. In the case of TfR recycling, recent evidence suggests myosin Vb most likely acts as a dynamic tether for Rab11-positive vesicles that reach the periphery, confining them to cortical actin and preventing them from moving back towards the cell centre on microtubules. In particular, elegant studies using a chemical-genetic approach to rapidly induce the stable binding of myosin Vb to actin showed that under these conditions recycling vesicles were trapped at the cell cortex. In contrast, when myosin Vb was prevented from binding to actin, Rab11 was trapped in the perinuclear region [154]. Recent studies support the dynamic tether model, but indicate that this may happen throughout the cell, not just at the cortex [155]. Interestingly, myosin V may not only regulate the return of recycled material to the plasma membrane, but might also contribute to the release of newly formed endocytic vesicles from the cortical actin. Disruption of myosin Vb impairs transit of newly internalised Tf from the cell periphery [156], whilst cells from myosin Va *null* mice display accelerated delivery of fluid-phase content to acidified endosomes [157]. Furthermore, Myosin Vb contributes to a complex (CART) that also contains the early-acting ESCRT-0 component Hrs and which contributes to a fast recycling pathway [158].

## 8 Conclusions and significance

The cytoskeleton and motors are essential for maintaining the spatial distribution of endocytic compartments within cells, and act at crucial points to facilitate transport steps such as membrane remodelling, carrier formation and cargo sorting. Paradoxically, experimental conditions that disrupt the positioning and motility of membranes profoundly are often of only minor consequence for the overall efficiency of endocytic functions such as receptor recycling or lysosomal delivery, at least in cultured cell models. This suggests that other transport steps are rate limiting in these cells, where distances between compartments are generally short, and indeed are often reduced by interventions such as microtubule motor disruption. In physiological systems, however, the movement and spatial distribution of endosomes is likely to be finely tuned and critical for cellular function. This is most clearly the case in highly polarised cells such as neurons, where the movement of endosomes and/or lysosomes is essential for processes such as dendritic branching [89], transport of survival factors [159], or protection against toxicity linked to neurodegeneration [103,160]. The control of endosome positioning is also crucial for spatially complex processes such as asymmetric cell division during embryogenesis [161] and tissue development [162], and the directed recycling of plasma membrane components during cell migration [163]. Since endosomes also act as platforms to maintain the distribution of other cellular constituents such as ribonucleoproteins [164,165], defects in their cellular distribution most likely impinge on an array of other cellular pathways.

## Figures and Tables

**Table 1:** Motor proteins and endocytic trafficking.

Motor/subunit	Organelle	Adaptor molecule	References
Kinesin-1			
Kif5	EE	Unknown	[64,105]
	LE/lysosomes	SKIP-Arl8	[64,111–113]
	Rab4/11 RE	Gadkin-AP1	[134]
	EE tubules; SNX1 & 8	Unknown	[58]

Kinesin-2			
Kif3A-Kif3B	Glut4 RE	Rab4	[166]
	RE	Rip11/FIP5	[133]
	LE/lysosomes	Unknown	[65,109]
	EE tubules; SNX4	Unknown	[58]

Kinesin-3			
KIF1Bβ	LE/lysosomes	Unknown	[115,135]
KIF13A	RE	Rab11	[132]
KIF16B	EE/RE	PtdIns-3P	[107,109]
Khc-73 (*D. melanogaster*)	EE	(Unknown)	[106]

Kinesin-13			
KIF2β	LE/lysosomes	Unknown	[116]

Kinesin-14			
KIFC1	EE	Unknown	[67]
KIFC2	EE	Unknown	[66]

Dynein/dynactin			
LIC1	LE/lysosomes	RILP	[63]
LIC1/2	RE	FIP3	[130,131]
LIC1	RE	Rab4	[90]
LIC (*D. rerio*)	LE/lysosomes	JIP3 (in neurons)	[100]
IC	LE/lysosomes	Snapin	[102]
LC8	EE tubules; SNX4	Kibra	[129]
p150^Glued^	LE/lysosomes	RILP/ORP1L	[96,97]
p150^Glued^	EE tubules; SNX1/2	SNX5/6	[128]
p25	EE	Hook	[92–95]

Myosins			
Myosin 1e	Coated pit	Dynamin, synaptojanin	[26]
Myosin 1b	EE	Unknown	[142–144]
Myosin Va	Endocytic vesicle	Unknown	[157]
Myosin Vb	RE	Rab11, Rab11-FIP2	[152,153]
Myosin Vb	RE	Hrs, CART	[158]
Myosin VI	Coated vesicle	DAB2	[32,33]
	Endocytic vesicle	GIPC, Tom1	[35,39]
	EE to RE	LMTK2	[141]
